# *Limosilactobacillus reuteri* ZY15 Alleviates Intestinal Inflammation and Barrier Dysfunction via AKT/mTOR/HIF-1α/RORγt/IL-17 Signaling and the Gut Microbiota in ETEC K88-Challenged *Mice*

**DOI:** 10.3390/antiox14010058

**Published:** 2025-01-06

**Authors:** Xin Xu, Hongwei Zhang, Kun Meng, Hongying Cai, Weiwei Liu, Liye Song, Zihan Zhang, Qijun Zhu, Xiling Han, Yunsheng Han, Peilong Yang

**Affiliations:** 1 Key Laboratory of Feed Biotechnology of Ministry of Agriculture and Rural Affairs, Institute of Feed Research, Chinese Academy of Agricultural Sciences, Beijing 100081, China; 82101221601@caas.cn (X.X.); mengkun@caas.cn (K.M.); caihongying@caas.cn (H.C.); liuweiwei01@caas.cn (W.L.); 82101231601@caas.cn (L.S.); zz0754@stu.syau.edu.cn (Z.Z.); 82101222008@caas.cn (Q.Z.); 2230120017@ynnu.edu.cn (X.H.); 2 Chengde Academy of Agriculture and Forestry Sciences, Chengde 067000, China; nkyzhanghongwei@126.com

**Keywords:** *Limosilactobacillus reuteri*, intestinal barrier, inflammation, gut microbiota, oxidative stress, IL-17

## Abstract

*Limosilactobacillus reuteri*, a recognized probiotic, improves intestinal health in animals, but the mechanism remains unclear. This study investigates the mechanisms by which *L. reuteri* ZY15, isolated from healthy pig feces, mitigates intestinal barrier damage and inflammation caused by oxidative stress in Enterotoxigenic *Escherichia coli* (ETEC) K88-challenged mice. The results indicated that *L. reuteri* ZY15 increased antioxidant capacity by reducing serum reactive oxygen species (ROS) and superoxide dismutase (SOD) levels. *L. reuteri* ZY15 enhanced the intestinal barrier by upregulating mucin 1, mucin 2, *occludin*, *zonula occludens-1* (*ZO-1*), and *claudin-1* expressions in protein and mRNA levels. It significantly alleviated intestinal inflammation by reducing the proinflammatory cytokines *interleukin-1β* (*IL-1β*), *interferon-γ* (*IFN-γ*), tumor necrosis factor-α (TNF-α), and interleukin-17 (IL-17) mRNA and protein levels. Notably, *L. reuteri* ZY15 suppressed intestinal inflammation by inhibiting AKT/mTOR/HIF-1α/RORγt/IL-17 pathway activation. Additionally, it significantly altered the structure of gut microorganisms by enriching *Akkermansia* and *Clostridia_UCG.014*, and thereby re-establishing colonization resistance and alleviating ETEC K88-induced intestinal barrier damage and inflammation in mice. Taken together, our findings reveal the protective mechanism of *L. reuteri* ZY15 in mice challenged with ETEC K88 by regulating AKT/mTOR/HIF-1α/RORγt/IL-17 signaling and microbial imbalance. Leveraging these properties, live *L. reuteri* ZY15 offers a promising alternative treatment for *Escherichia coli*-induced diarrhea in weaned piglets.

## 1. Introduction

In modern swine breeding, weaning is a critical period in a pig’s life [1]. Antibiotics overuse is closely related to a growing number of antimicrobial-resistant agents and raises important concerns about animal welfare [2]. Additionally, some bacterial families such as *Enterobacteriaceae* are resistant to nearly all antibiotics, including last-resort drugs [1]. During weaning transition, gastrointestinal infections mainly occur, including colibacillosis diarrhea [3]. These negative outcomes have prompted researchers to seek antibiotic alternatives to maintain both piglet health at critical weaning periods and also preserve public health. Currently, biocontrol strategies using probiotics to suppress pathogens are accepted, viable approaches to combatting bacterial gut infections [4].

The intestine is the largest digestive and immune organ in piglets, with intestinal health crucial for healthy growth and development [5]. The intestinal epithelium functions as a barrier between the external environment and the closely regulated internal milieu and is essential for host health [6]. Intestinal inflammatory responses leading to mucosal barrier damage are key processes underlying diarrhea in piglets [7]. In recent years, studies have shown that intestinal inflammation is closely related to tissue hypoxia [8]. Under such conditions, innate immune cells of the lamina propria undergo acute inflammatory responses, leading to considerable inflammatory cytokine production [9]. For example, helper T cells (Th17), which are associated with inflammatory and autoimmune disease pathogenesis, produce high interleukin (IL)-17 levels [10]. IL-17 has a dominant role in the inflammatory network and is associated with periodontitis traits, suggesting that abnormal, IL-17-induced inflammatory responses may cause tissue damage [11]. Mechanistically, IL-17 appears to amplify inflammation by enhancing pro-inflammatory cytokine/chemokine production, leading to excessive neutrophil recruitment, which in turn activates osteoclasts and bone resorption [12]. Critically, intestinal commensal probiotics have key roles in maintaining host intestinal health [13]. Colonization by sufficient probiotic numbers in the gut inhibits pathogenic bacteria subsistence and proliferation, which prevents intestinal mucosal barrier disruption [14]. A pathogen-disturbed gut microbiota shows impaired function, with damage to the distal organ and tissue homeostasis occurring via gut-targeted signaling axes [15].

*Limosilactobacillus reuteri* is a *Lactobacillus* which inhibits inflammation, strengthens intestinal barrier function, maintains intestinal regeneration, and repairs damaged intestinal mucosa [16]. Tao et al. reported that *Escherichia coli* was significantly enriched in diarrheic piglets and that *Lactobacillus* was the most prevalent bacterial community in healthy animal feces, with *L. john* and *L. reuteri* being highly abundant in *Lactobacillus* [17]. The targeted alteration of intestinal microbiota using probiotics has become a potential treatment option for intestinal illnesses [18]. *L. reuteri* kills pathogens in multiple ways, including the secretion of antibacterial substances such as organic acids, ethanol, and reuterin [19]. Yi et al. reported that *L. reuteri* LR1 exerted positive effects against Enterotoxigenic *E. coli* K88 (ETEC K88) infection in intestinal porcine epithelial cells 1 (IPEC-1 cells) [20]. However, research is lacking on how *L. reuteri* relieves intestinal inflammation and enhances the intestinal barrier [21]. Even within the same species, different *L. reuteri* strains show diverse biological traits [22]. For instance, in the digestive tract of animals with strong resistance to infection, some probiotics may promote pathogen tolerance [23]. Therefore, deriving probiotics from the digestive tracts of animals with strong resistance to infection and using them to clarify disease resistance mechanisms and explore their use in preventing intestinal diseases is a promising but challenging research avenue.

Mice are compelling and reliable biomedical models for investigating pig intestinal disorders [24]. In recent research (our unpublished data), highly abundant *L. reuteri* levels were identified in weaned piglets with high resistance to weaning stress, termed ZY15. In this study, ETEC K88 was used to induce intestinal inflammatory damage in C57BL/6 mice, and then an intervention was performed using *L. reuteri* ZY15. We identified key *L. reuteri* ZY15 effects on the intestinal barrier and inflammation and associated mechanisms by combining 16S *rRNA* gut microbiota analyses with transcriptomic techniques.

## 2. Materials and Methods

### 2.1. Mice and Bacteria

Seventy-two C57BL/6 mice (4-week-old males, 17 g ± 0.06 g) were purchased from Beijing Vital River Laboratory Animal Technology Co., Ltd. (Beijing, China). The mice were housed under a 12 h light/12 h dark cycle in standard specific-pathogen-free (SPF) conditions. The study protocol was approved by the Institutional Animal Care and Use Committee of the Institute of Feed Research of Chinese Academy of Agricultural Sciences (Approval number: IFR-CAAS20221101). The mice were given a common SPF diet (Vital River Laboratory Animal Technology Co., Ltd.) and free access to water. *L. reuteri* ZY15 (CGMCC NO.28937) was cultured in De ManRogosa Sharpe medium (MRS) at 37 °C. ETEC K88 was cultured in Luria-Bertani medium at 37 °C and 200 rpm. After a week of acclimatization, the mice were randomly divided (*n* = 18 mice/group) into control (Con), ETEC K88 (K88), *L. reuteri* ZY15 (ZY15), and protected (ZY15-K88) groups. From day 1 to 21, the Con and K88 mice were given 200 µL of sterile MRS, while the ZY15 and ZY15-K88 mice received 200 µL of *L. reuteri* ZY15 (10^9^ colony-forming units (CFU)/mL [23]). From day 15 to 21, on the basis of the front step, Con and ZY15 mice received 200 µL of sterile LB, while K88 and ZY15-K88 mice received 200 µL of 10^9^ ETEC K88 CFU/mL.

### 2.2. Sample Collection

The mouse body weights were recorded weekly throughout the study. At the end of testing, the mice were anesthetized and cervically dislocated. Then, 5 mL of blood was collected, and after 2 h, the blood was centrifuged at 3000× *g* at 4 °C for 10 min to recover serum, which was stored at −20 °C. The samples included serum, feces, chyme, duodenum, jejunum, and ileum. The intestines were divided into four sections: duodenum, jejunum, ileum, and colon. The tissues were rapidly washed in saline, fixed in 4% formalin, and stored at 4 °C. Intestinal tissue samples were stained with hematoxylin & eosin (H&E) and periodic acid–Schiff stains. All tissue samples were washed in phosphate-buffered saline (PBS), rapidly frozen in liquid nitrogen, and stored at −80 °C.

### 2.3. Morphology and Histology Analysis

Ileum tissues in 4% paraformaldehyde were trimmed, dehydrated, and embedded in paraffin for sectioning. Wax blocks were cut into 5 μm standard transverse sections, stained with H&E, and sealed with cedar oil. Images were obtained using a BX53 microscope 269 (Olympus, Tokyo, Japan). Three intact crypt villi at three typical locations per sample were blindly examined to measure the villus height and adjoining crypt depth and calculate ratios. A histological score system was applied: 0–30 for each parameter [25].

### 2.4. Lactobacillus and E. coli Load Detection in Colonic Chyme and Visceral Organs

Feces were added to normal sterile saline at 0.1 g/mL. After homogenizing with 1 mm sterile steel balls, the mixtures were serially diluted to 1 × 10^6^ and 100 μL and spread onto Eosin Methylene Blue (EMB) and MRS agar plates using sterile glass beads. After aerobic culture for approximately 18 h at 37 °C, viable white positive colonies were counted, ranging from 30 to 300/plate. Accordingly, *Lactobacillus* and *E. coli* loads were determined.

### 2.5. Real-Time Quantitative PCR

Total RNA was extracted from the ileum samples using an RNA extraction kit (TIANGEN Biotechnology Co., Ltd., Beijing, China). The total RNA concentrations and quality were determined using a NanoDrop 2000 spectrophotometer (Thermo Fisher Scientific, Waltham, MA, USA), which confirmed high-quality absorbance ratios (260/280 nm) between 1.8 and 2.0. Reverse transcription to cDNA was conducted using 1 µg RNA (M5-Super qPCR RT kit with gDNA remover, Beijing Mei5 Biotechnology Co., Ltd., Beijing, China). Cytokine expression in the ileum was determined by SYBR green real-time PCR using a Light Cycler^®^ 96 instrument (Roche, Mannheim, Germany). Primer sequences are shown (Table 1). The 2^−ΔΔCt^ quantification method was used, with *GAPDH* as a reference gene, and relative expression was normalized to Con values.

### 2.6. Enzyme-Linked Immunosorbent Assay

Ileal tissue was washed in cold PBS (pH 7.4), after which 0.1 g was homogenized in 0.9 mL PBS. Then, the homogenates were centrifuged at 3000× *g* for 15 min at 4 °C (Hirachi Koki Co., Ltd., Tokyo, Japan). The supernatants were harvested to determine lipopolysaccharide (LPS), diamine oxidase (DAO), reactive oxygen species (ROS), superoxide dismutase (SOD), tumor necrosis factor-α (TNF-α), mucin 1 (MUC1), mucin 2 (MUC2), transforming growth protein kinase B (AKT), mammalian target of rapamycin complex1 (mTORC1), mammalian target of rapamycin complex2 (mTORC2), hypoxia-inducible factor (HIF-1α), retinoid-related orphan receptor gamma t (RORγt), and IL-17 levels using ELISA kits (Shanghai Enzyme-linked Biotechnology Co., Ltd., Shanghai, China) according to the kit instructions on a microplate reader (Thermo Fisher Scientific, Vantaa, Finland). Parameters from six random mice/group were measured.

### 2.7. Transcriptomics

Sequencing was performed by Beijing Novogene Co., Ltd. (Beijing, China). RNA extraction mini kits (Qiagen, Hilden, Germany) were used to extract total RNA from the ileum samples, and a NanoDrop 2000 instrument used for RNA quantification. Library construction and quality control steps were performed and raw RNA-sequencing data were filtered. After cluster generation, the libraries were sequenced on an Illumina Novaseq platform and 150 bp paired-end reads were generated. Differential expression analysis of two conditions/group was performed using the DESeq2 R package (v.1.20.0), which provided statistical analyses to determine differential expression in digital gene expression data using a negative binomial distribution model. *p*-values were adjusted using Benjamini and Hochberg’s false discovery rates. Genes with adjusted *p*-values < 0.05 (by DESeq2) were assigned as differentially expressed genes (DEGs), with volcano plots used for DEG visualization. Gene Ontology (GO) enrichment analysis of DEGs was conducted in the cluster Profiler R package, where gene length bias was corrected. GO terms with corrected *p*-values < 0.05 were deemed significantly enriched DEGs.

### 2.8. 16S rRNA Gut Microbiota Analysis

Total genomic DNA from cecal chyme was extracted using Stool DNA Kits (TianGen, China). Then, *16S rRNA* V3–V4 regions were amplified using 341F: 5′-CCTAYGGGRBGCASCAG-3′ 806R: 5′-GGACTACNNGGGTATCTAAT-3′ primer-containing barcodes. All resultant amplicons were equally mixed. Sequencing libraries were generated using New England Biolabs (NEB) Next^®^ Ultra™ II FS DNA PCR-free Library Prep Kits (New England Biolabs, Ipswich, MA, USA), after which indexes were added following the manufacturer’s recommendations. The library was checked with Qubit and real-time PCR for quantification and a bioanalyzer for size distribution detection. The quantified libraries were then pooled and sequenced on Illumina platforms, based on effective library concentrations and the amount of data required. For previously obtained Effective Tags, denoising was performed using DADA2 or the deblur module in QIIME2 software (v.QIIME2-202006) to generate initial Amplicon Sequence Variants (ASVs) (default: DADA2); ASVs with an abundance < 5 were filtered out. To analyze bacterial community diversity, richness, and uniformity in samples, QIIME2 was used to calculate α-diversity from Chao1 and Shannon indices. To evaluate community composition complexity and compare differences between samples (groups), principal coordinates analysis (PCoA) and nonmetric multidimensional scaling methods were used to analyze β-diversity. T-tests and two-way analysis of variance (ANOVA) were used to compare microbiota differences between groups.

### 2.9. Statistical Analysis

Data were analyzed using ANOVA with general linear model procedures in SPSS 26.0 (IBM Corp., Armonk, NY, USA) in a 2 × 2 factorial design. The statistical model included dietary effects (MRS or *L. reuteri* ZY15), challenge (LB or *E. coli* K88), and interactions. When a significant interaction trend was observed, data were further analyzed using one-way ANOVA with Duncan’s multiple range tests. Graphs were assembled in GraphPad Prism (v.9.0, GraphPad Software, San Diego, CA, USA) and data were represented by the mean ± standard error (*n* = 6 mice/group). *p* < 0.05 and *p* < 0.01 values were considered significant and extremely significant, respectively, whereas 0.05 < *p* < 0.10 indicated a tendency.

## 3. Results

### 3.1. L. reuteri ZY15 Effects on Mouse Body Weight, Serum Indices, and Fecal Bacterial Composition After ETEC K88 Challenge

**Figure 1:**
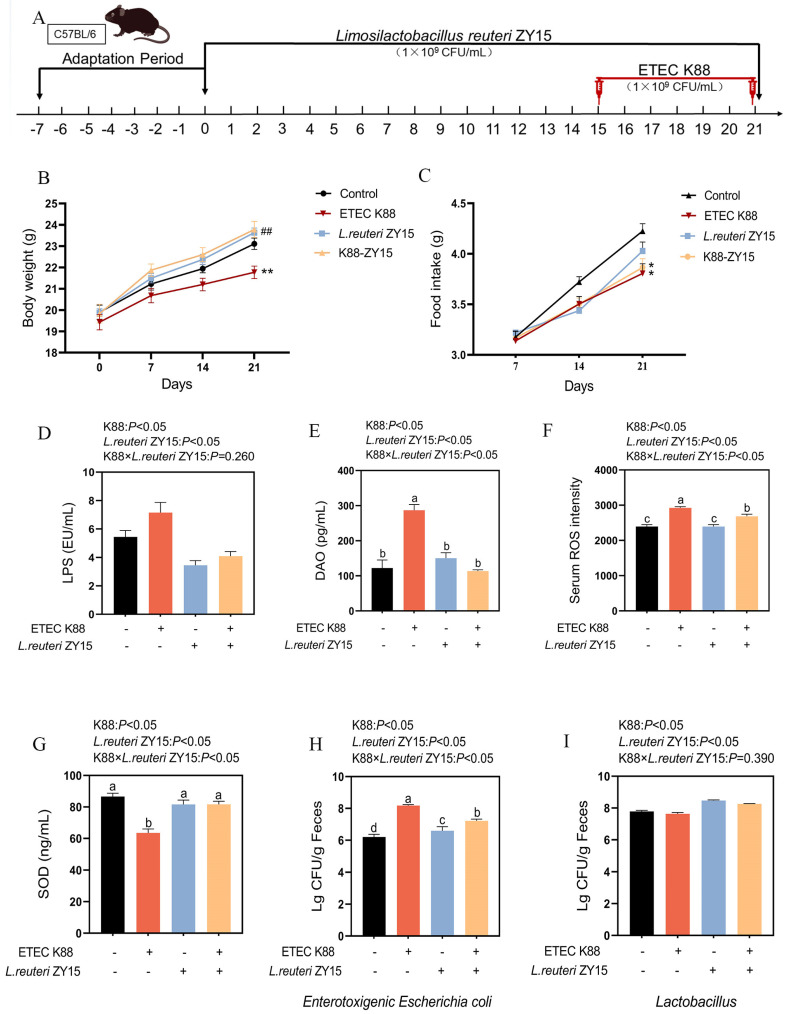
*L. reuteri* ZY15 effects on body weight, serum indices, and bacterial composition in feces after ETEC K88 challenge. (**A**) The experimental design and groupings; (**B**) Body weights; (**C**) Food intake; (**D**) Serum lipopolysaccharide (LPS) levels; (**E**) Serum diamine oxidase (DAO) concentrations; (**F**) Serum reactive oxygen species (ROS) concentrations; (**G**) Serum superoxide dismutase (SOD) concentrations. (**H**,**I**) Fecal coliform and lactic acid bacterial numbers on day 21. Data are expressed as the mean ± standard error (*n* = 6). * *p* < 0.05, ** *p* < 0.01 vs. control group; ^##^ *p* < 0.01 vs. K88 group. (**D**–**I**) Differences among treatments were analyzed by two-way ANOVA. ^a–d^ represent different significant differences (K88 × *L. reuteri* ZY15: *p* < 0.05). K88 indicates dietary supplementation with ETEC K88 or not; *L. reuteri* ZY15 indicates the dietary supplementation of *L. reuteri* ZY15 or not.

From day 1 to 7, the body weights across groups showed similar, gradual increases (*p* > 0.05, Figure 1). From day 8 to 14, the body weights in Con and K88 mice increased gradually, but on day 14 in ZY15 and ZY15-K88 mice, the body weights significantly increased when compared with Con and K88 mice (*p* < 0.05). However, from day 15, body weights in K88 mice decreased significantly when compared with Con mice (*p* < 0.05, Figure 1B). Also, when compared with Con mice, the food intake in K88 and ZY15-K88 mice decreased significantly from day 15 (*p* < 0.05, Figure 1C). These observations in ZY15 and ZY15-K88 mice suggested that ZY15 exerted better remission effects on K88.

No interaction between ZY15 dietary treatment and K88 challenge was found for serum LPS levels (*p* > 0.05, Figure 1D). When compared with Con mice, K88-challenged mice showed significantly increased serum LPS levels, while *L. reuteri* ZY15 significantly decreased these levels (*p* < 0.05). Differences between the dietary treatment and ETEC K88-challenged mice were identified for serum DAO, ROS, and SOD levels (*p* < 0.05, Figure 1E–G), with higher values in K88 mice when compared with Con, ZY15, and ZY15-K88 animals (*p* < 0.05). The serum ROS levels in ZY15-K88 mice were higher than those in ZY15 mice (*p* < 0.05), but no significant differences in serum DAO and SOD levels were identified between ZY15-K88 and ZY15 animals (*p* > 0.05).

An interaction in fecal coliform numbers on day 21 was identified, with higher numbers in K88 mice when compared with Con, ZY15, and ZY15-K88 animals (*p* < 0.05, Figure 1H). When compared with Con mice, ETEC K88-challenged mice showed significantly decreased fecal lactic acid bacterial numbers, but *L. reuteri* ZY15 significantly reversed this (*p* < 0.05, Figure 1I). On day 21, no interaction in fecal lactic acid bacterial numbers was identified between dietary treatment and ETEC K88-challenged mice (*p* > 0.05).

### 3.2. L. reuteri ZY15 Effects on Intestinal Pathology and Morphology After ETEC K88 Challenge

**Figure 2:**
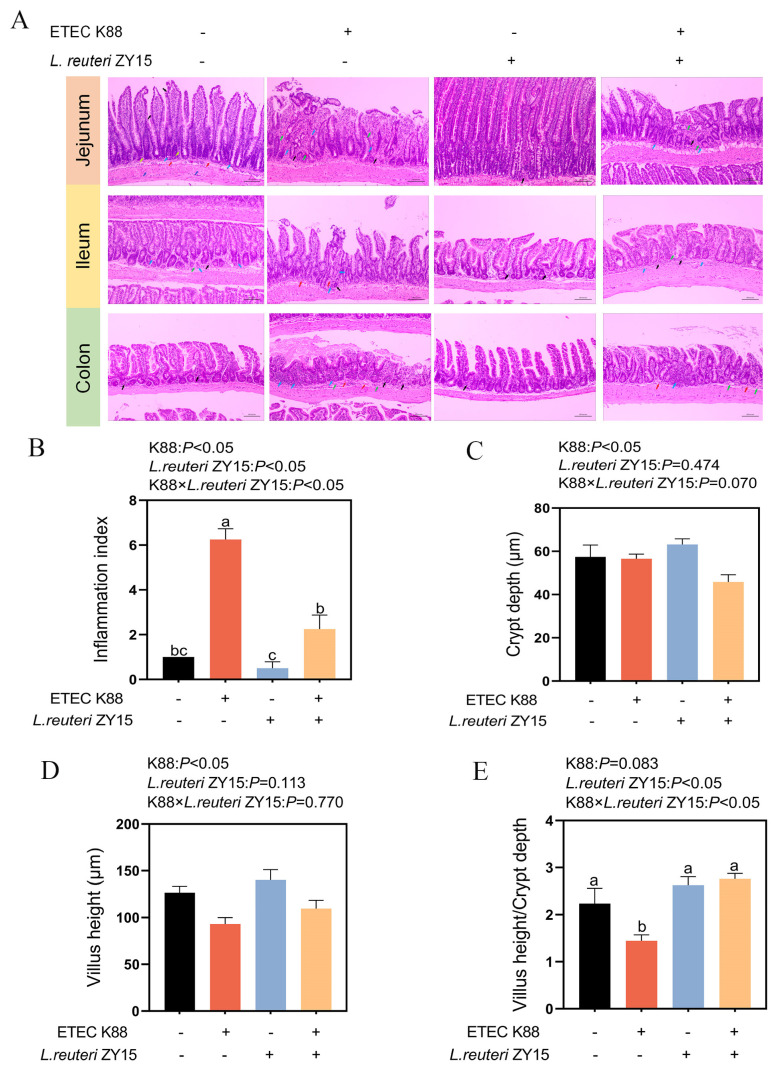
*L. reuteri* ZY15 effects on intestinal tissue pathology and morphology after ETEC challenge. (**A**) Representative jejunum, ileum, and colon histological sections (bar 200 microns). Deep neutrophil and monocyte infiltration into serosal layers (blue arrows), local mucosal erosion and crypt disappearance (black arrows), mucosal lamina propria angiogenesis (red arrows), and fibrous tissue propria at significant inflammation sites (green arrows); (**B**) ileal crypt depth; (**C**) ileal villus height; (**D**) villus-height-to-ileum-crypt-depth ratios; (**E**) pathological scores in the ileum. (**B**–**E**) Data are expressed as the mean ± standard error (*n* = 6). Differences among treatments were analyzed by two-way ANOVA. ^a–c^ represent different significant differences (K88 ×*L. reuteri* ZY15: *p* < 0.05). K88 indicates dietary supplementation with ETEC K88 or not; *L. reuteri* ZY15 indicates the dietary supplementation of *L. reuteri* ZY15 or not.

ETEC K88 challenge generated severe colon, jejunal, and ileal tissue damage, including deep neutrophil and monocyte infiltration into serosal layers (blue arrows), local mucosal erosion and crypt disappearance (black arrows), mucosal lamina propria angiogenesis (red arrows), and fibrous tissue propria at significant inflammation sites (green arrows) (Figure 2A). These findings were consistent with pathological scores for the ileum, where an interaction between dietary treatment and ETEC K88 challenge was discovered (*p* < 0.05, Figure 2B). Mice fed *L. reuteri* ZY15 had lower pathological scores when compared with ETEC K88-challenged mice on a basal diet (*p* < 0.05). Additionally, when compared with Con and ZY15 mice, ETEC-challenged mice showed significantly decreased ileal villus heights (*p* < 0.05, Figure 2D); an interaction between dietary treatment and ETEC K88 challenge was determined for villus-height-to-crypt-depth (V/C) ratios in the ileum (*p* < 0.05, Figure 2E), and an interaction trend was identified for ileal crypt depth (0.01 < *p* < 0.05, Figure 2C). When compared with ETEC K88-challenged mice on a basal diet, mice receiving *L. reuteri* ZY15 showed significantly increased V/C values (*p* < 0.05) and a decreased trend in crypt depth (0.01 < *p* < 0.05).

### 3.3. L. reuteri ZY15 Effects on the Ileal Transcriptome After ETEC K88 Challenge

**Figure 3:**
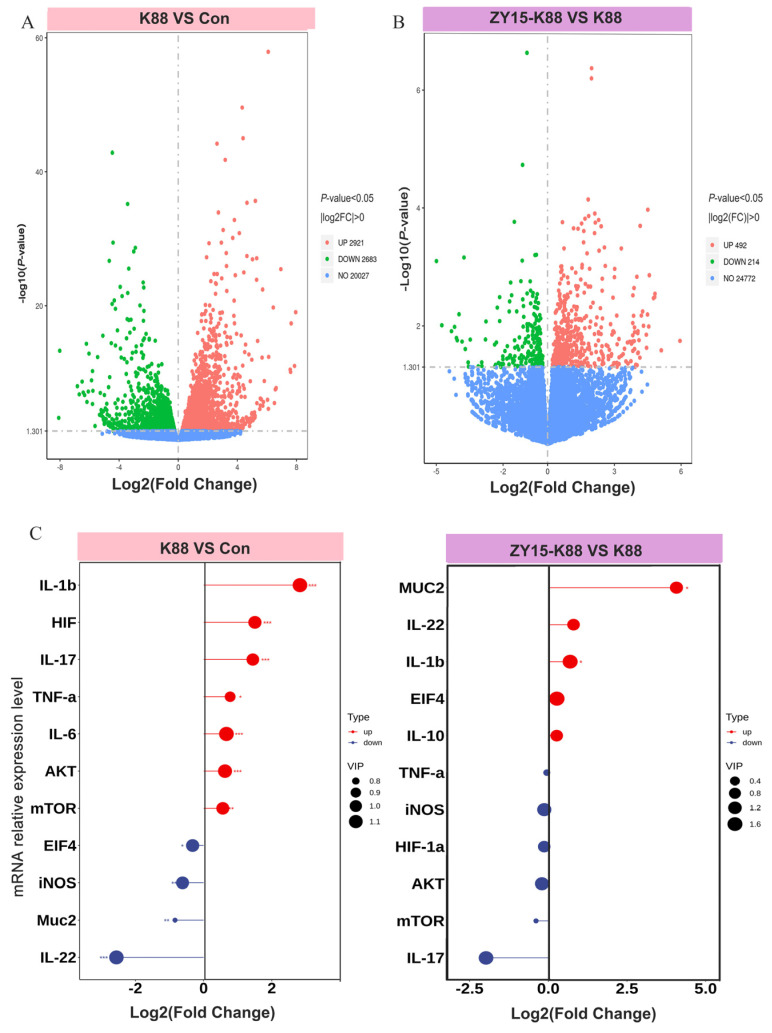
Differentially expressed gene (DEG) analyses in the ileum after ETEC challenge. (**A**) Volcano plots showing DEGs in Con and K88 groups. A total of 2921 upregulated and 2683 downregulated DEGs were identified between the K88 and Con groups. (**B**) Volcano plots showing DEGs in K88 and ZY15–K88 groups. A total of 492 upregulated and 214 downregulated DEGs were identified between the ZY15–K88 and the K88 groups. (**C**) Specific mRNA gene expression in Con and K88 and K88 and ZY15–K88 samples. *t*-tests were used in analyses. Differences are significant at * *p* < 0.05; ** *p* < 0.01; and *** *p* < 0.001 (*n* = 4). Con, basal diet group; ZY15, basal diet group (*L. reuteri* ZY15 diet group); K88, basal diet group treated with ETEC K88; ZY15–K88, *L. reuteri* ZY15 diet group treated with ETEC K88.

Volcano plots showed that ZY15 significantly affected DEG distribution. In total, 2921 genes were upregulated (red dots above the dashed line), while 2683 genes were downregulated (green dots below the dashed line) in K88 mice when compared with CON mice (Figure 3A). The ZY15-K88 v. K88 comparison also showed distinct DEG pattern changes. In total, 492 genes were upregulated and 214 downregulated in ZY15-K88 mice relative to K88 mice (Figure 3B).

Ileal transcriptome analyses showed that *MUC2* expression was significantly reduced in K88 samples but significantly increased in ZY15-K88 samples (*p* < 0.05) (Figure 3C). Inflammatory pathway gene analyses showed that ZY15 also inhibited *TNF-α*, *HIF-1α*, *IL-17*, *AKT*, and *mTOR* mRNA expression (*p* > 0.05). *HIF-1α*, *AKT*, and *mTOR* are signaling genes in the AKT-mTOR signaling pathway (Figure 3C).

**Figure 4:**
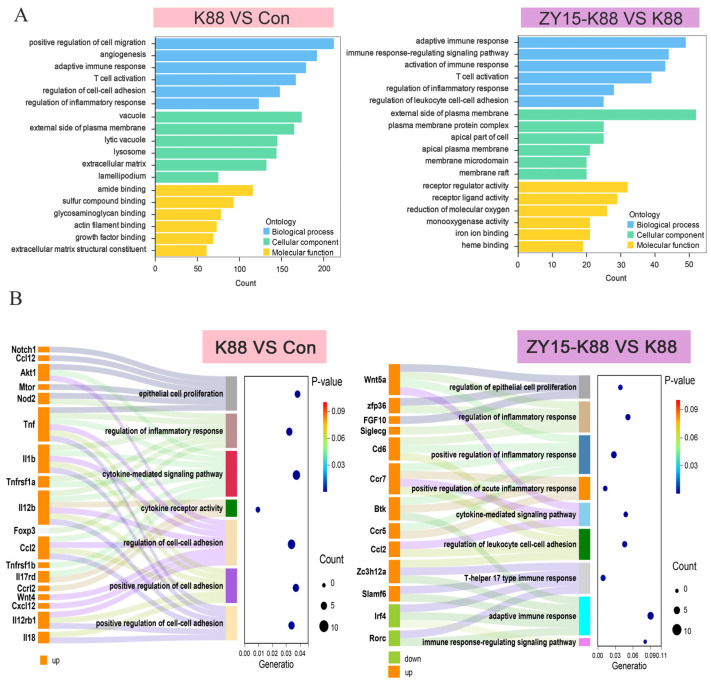
Gene Ontology (GO) pathway analyses of differentially expressed genes (DEGs) in the ileum after ETEC challenge. (**A**) GO pathway analyses of DEGs in Con and K88 and K88 and ZY15–K88 samples. (**B**) GO–BP analysis of significantl DEGs in Con and K88 and K88 and ZY15–K88 samples. Groups had at least four biological replicates. Con, basal diet group; ZY15, basal diet group (*L. reuteri* ZY15 diet group); K88, basal diet group treated with ETEC K88; and ZY15-K88, *L. reuteri* ZY15 diet group treated with ETEC K88.

GO results are categorized into three main domains: biological processes, cellular components, and molecular functions. GO analyses showed that when compared with Con samples, K88 samples were mainly enriched for five inflammation- and four energy metabolism-related pathways (Figure 4A). When compared with K88 samples, ZY15-K88 samples were mainly enriched for six inflammation- and five energy metabolism-related pathways (Figure 4A), underscoring a potential enhancement in adaptive immune mechanisms in response to ZY15 treatment. Considering that the DEGs have the largest proportion of participation in biological processes (Figure 4A), we further conducted a GO biological process (GO-BP) analysis (*n* = 4) on significantly upregulated DEGs. The results of the GO-BP analysis showed that when compared with Con samples, significantly regulated DEGs in K88 samples were mainly enriched for cytokine-mediated signaling, the positive regulation of cell adhesion, and five other GO pathways. When compared with K88 samples, significantly regulated DEGs in ZY15-K88 samples were mainly enriched for regulated inflammatory responses, T-helper 17 type immune responses, cytokine-mediated signaling, and four other GO pathways (Figure 4B).

### 3.4. L. reuteri ZY15 Effects on Ileal Inflammation and Barrier Function Integrity After ETEC K88 Challenge

**Figure 5:**
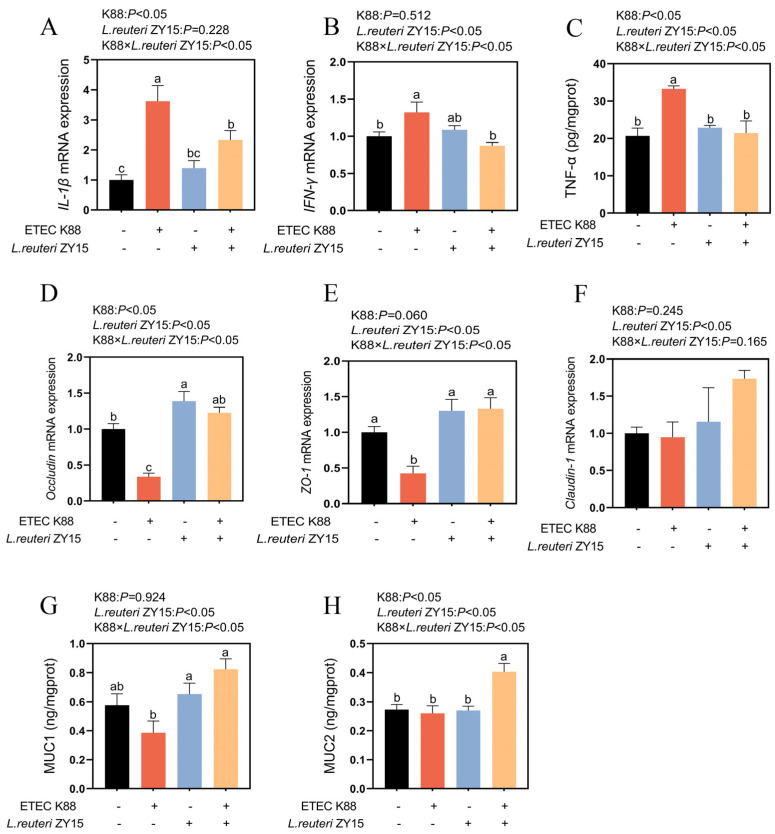
*L. reuteri* ZY15 effects on ileal inflammation and barrier function after ETEC K88 challenge. (**A**,**B**) Ileal *IL-1β* and *IFN-γ* mRNA expression levels. (**C**) Ileal TNF-α expression levels. (**D**–**F**) *Occludin*, *ZO-1*, and *Claudin-1* mRNA expression levels in ileum tissue. (**G**,**H**) MUC1 and MUC2 expression levels in ileum tissue. Data are expressed as the mean ± standard error (*n* = 6). Differences among treatments were analyzed by two-way ANOVA. ^a–c^ represent different significant differences (K88 × *L. reuteri* ZY15: *p* < 0.05). K88 indicates dietary supplementation with ETEC K88 or not; *L. reuteri* ZY15 indicates the dietary supplementation of *L. reuteri* ZY15 or not.

An interaction was identified between dietary treatment and ETEC K88 challenge samples for *IL-1β*, *TNF-α*, *IFN-γ*, *Occludin*, *ZO-1*, *MUC1*, and *MUC2* mRNA expression levels (*p* < 0.05, Figure 5), but no interaction was identified for *Claudin-1* mRNA expression (*p* > 0.05). When compared with Con samples, *IL-1β*, *TNF-α*, and *IFN-γ* mRNA expression levels in K88 samples were significantly upregulated, but *Occludin* and *ZO-1* mRNA expression levels were significantly downregulated (*p* < 0.05). Mice fed *L. reuteri* ZY15 showed lower ileal *IL-1β*, *TNF-α*, and *IFN-γ* mRNA expression levels, whereas higher *Occludin*, *ZO-1*, *MUC1*, and *MUC2* mRNA expression levels were identified when compared with ETEC K88-challenged mice on a basal diet (*p* < 0.05). Also, when compared with mice fed MRS medium, *L. reuteri* ZY15 significantly upregulated *Claudin-1* mRNA expression (*p* < 0.05).

### 3.5. L. reuteri ZY15 Effects on the AKT/mTOR/HIF-1α/RORγt/IL-17 Pathway in Ileum Tissue After ETEC K88 Challenge

**Figure 6:**
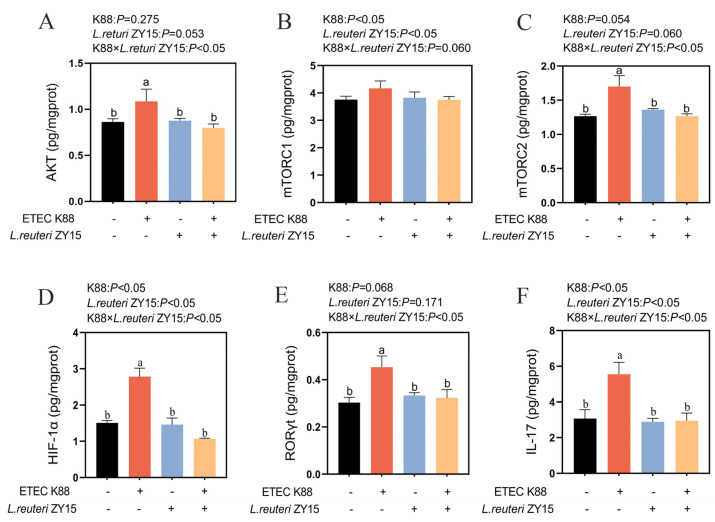
*L. reuteri* ZY15 effects on the AKT/mTOR/HIF-1α/RORγt/IL-17 pathway in ileum tissue after ETEC K88 challenge. (**A**−**F**) Ileal AKT, mTORC1, mTORC2, HIF-1α, RORγt, and IL-17 expression levels. Data are expressed as the mean ± standard error (*n* = 6). Differences among treatments were analyzed by two-way ANOVA. ^a,b^ represent different significant differences (K88 ×*L. reuteri* ZY15: *p* < 0.05). K88 indicates dietary supplementation with ETEC K88 or not; *L. reuteri* ZY15 indicates the dietary supplementation of *L. reuteri* ZY15 or not.

An interaction was identified between dietary treatment and ETEC K88 challenge for AKT, mTORC2, HIF-1α, RORγt, and IL-17 expression levels (*p* < 0.05, Figure 6A–F), while an interaction trend was identified for mTORC1 (0.05 < *p* < 0.1, Figure 6B). When compared with Con mice, ileal AKT, mTORC2, HIF-1α, RORγt, and IL-17 levels in K88 animals were significantly increased (*p* < 0.05), while *L. reuteri* ZY15 levels significantly decreased when compared with those of K88 mice (*p* < 0.05). *L. reuteri* ZY15 reversed the mTORC1 trend by upregulating the expression levels induced by K88 challenge (0.05 < *p* < 0.1).

### 3.6. L. reuteri ZY15 Effects on Bacterial Composition in Cecal Digesta After ETEC K88 Challenge

**Figure 7:**
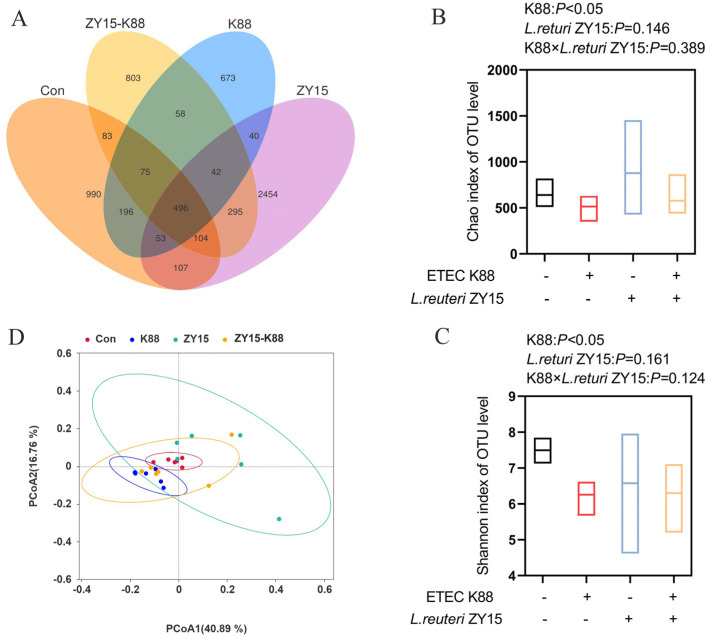
*L. reuteri* ZY15 effects on cecal microbiota diversity after ETEC K88 challenge. (**A**) Venn diagram showing gut microbiome ASVs across groups. (**B**) Chao α-diversity index. (**C**) Shannon α-diversity index. (**D**) PCoA distance at species levels. (**B**,**C**) Data are expressed as the mean ± standard error (*n* = 6). Differences among treatments were analyzed by two-way ANOVA. K88 indicates dietary supplementation with ETEC K88 or not; *L. reuteri* ZY15 indicates the dietary supplementation of *L. reuteri* ZY15 or not.

In total, 6469 ASVs were observed. Venn diagram analysis (Figure 7A) showed a 496 ASV overlap across groups, which indicated consistent, common core species across groups. However, analyses also provided information about unique species, which differed across groups, with 990, 673, 2454, and 803 unique ASVs identified in the Con, K88, ZY15, and ZY15-K88 groups, respectively. Alpha-diversity analysis showed that when compared with Con mice, K88-challenged mice showed significantly decreased Chao and Shannon indices (*p* < 0.05, Figure 7B,C), but *L. reuteri* ZY15 exerted no obvious effects on bacterial richness and diversity (*p* > 0.05). No interaction was identified between dietary treatment and ETEC K88 challenge in terms of α-diversity. Also, β-diversity analysis revealed clear microbial composition segregation and dissimilarities across all groups (Figure 7D).

**Figure 8:**
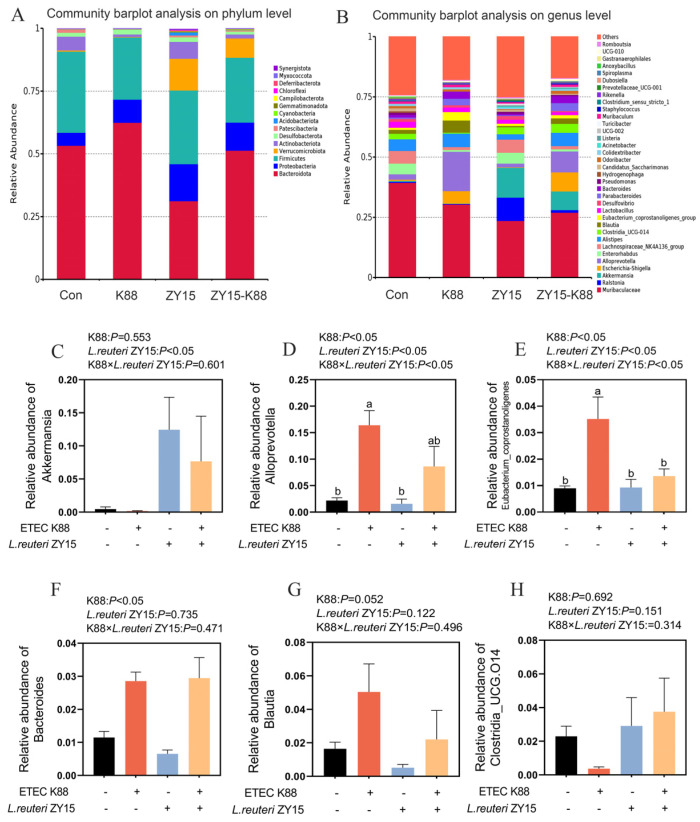
*L. reuteri* ZY15 effects on community abundance at phylum and genus levels in cecal microbiota after ETEC K88 challenge. (**A**) Top 15 relatively abundant microorganisms at phylum levels. (**B**) Top 35 relatively abundant microorganisms at genus levels. (**C**–**H**) Community abundance of *Akkermansia* (**C**), *Alloprevotella* (**D**), *Eubacterium_coprostanoligenes_group* (**E**), *Bacteroides* (**F**), *Blautia* (**G**), and *Clostridia_UCG.014* (H) in cecal microbiota. Each group had at least six biological replicates. (**C**–**H**) Data are expressed as the mean ± standard error (*n* = 6). Differences among treatments were analyzed by two-way ANOVA. ^a,b^ represent different significant differences (K88 ×*L. reuteri* ZY15: *p* < 0.05). K88 indicates dietary supplementation with ETEC K88 or not; *L. reuteri* ZY15 indicates the dietary supplementation of *L. reuteri* ZY15 or not.

At phylum levels (Figure 8A), *Bacteroidota*, *Proteobacteria*, *Firmicutes*, *Verrucomicrobiota*, and *Actinobacteriota* were dominant across groups, with a total relative abundance of >95%. When compared with Con mice, K88 reduced relative *Firmicutes*, *Verrucomicrobiota*, *Actinobacteriota*, and *Patescibacteria* abundance and the *Firmicutes*-to-*Bacteroidota* (F/B) ratio, while being supplied with ZY15 reconstructed these microbes.

At genus levels (Figure 8B), when compared with Con mice, relative gut health-related bacterial abundance (e.g., *Akkermansia*, *Enterorhabdus*, *Turicibacter*, *Dubosiella*, and *Lachnospiraceae_NK4A136_group*) was decreased, while other bacteria (*Escherichia-Shigella*, *Eubacterium_coprostanoligenes_group*, and *Colidextribacter*) were increased in K88 mice, whereas ZY15 reconstructed these microbes. No differences between ZY15 dietary treatment and K88 challenge were identified for relative *Akkermansia* abundance (*p* > 0.05, Figure 8C). Relative *Akkermansia* abundance in ZY15 mice was higher than in Con and K88 mice (*p* < 0.05). Interactions between ZY15 dietary treatment and K88 challenge were identified for relative *Alloprevotella* and *Eubacterium_coprostanoligenes_group* abundance (*p* < 0.05, Figure 8D,E), with higher values in K88 mice when compared with Con, ZY15, and ZY15-K88 mice (*p* < 0.05). No interaction between dietary treatment and ETEC K88 challenge was identified for *Bacteroides*, *Blautia*, and *Clostridia_UCG.014* (*p* > 0.05, Figure 8F–H). When compared with Con mice, relative *Bacteroides* (*p* < 0.05) and *Blautia* (*p* > 0.05) abundance in K88 mice was increased, while *L. reuteri* ZY15 decreased those levels when compared with K88 mice (*p* > 0.05). When compared with Con mice, relative *Clostridia_UCG.014* abundance in K88 mice was decreased, while *L. reuteri* ZY15 increased these levels when compared with K88 mice (*p* > 0.05).

### 3.7. L. reuteri ZY15 Correlations Between the Gut Microbiota and Cytokine Levels After ETEC K88 Challenge

**Figure 9:**
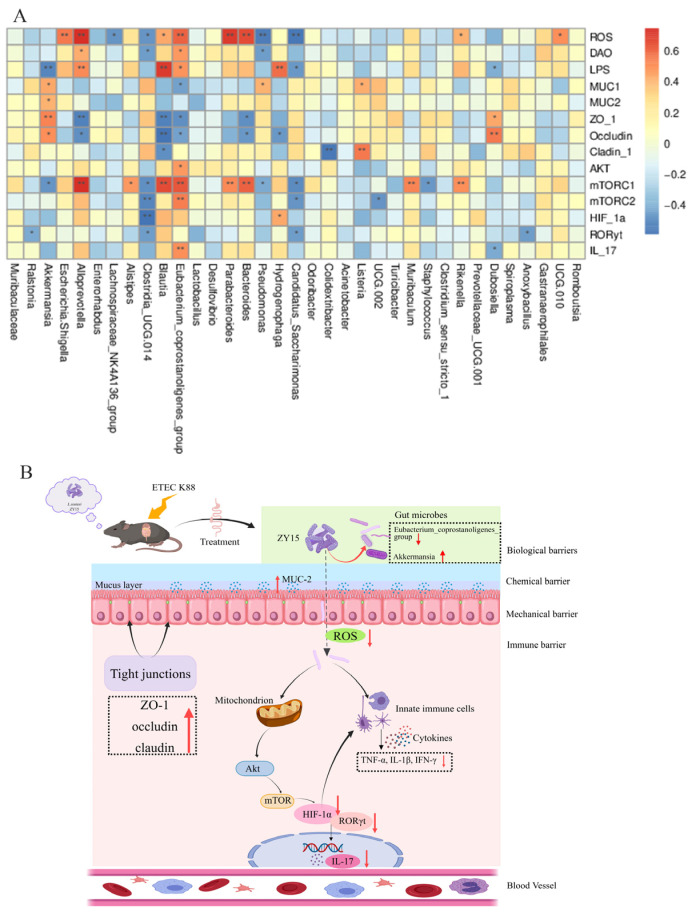
*L. reuteri* ZY15 correlations between the gut microbiota (top 35 phyla and genus levels) and cytokine levels after ETEC K88 challenge. (**A**) Spearman’s correlation heatmap showing genus levels. (**B**) Schematic showing how *L. reuteri* ZY15 improves inflammation at the intestines after ETEC K88 challenge. The red upward arrow indicates upregulation of gene expression, while the red downward arrow indicates downregulation of gene expression. * *p* < 0.05 and ** *p* < 0.01 (*n* = 6). Con, basal diet group; ZY15, basal diet group (*L. reuteri* ZY15 diet group); K88, basal diet group treated with ETEC K88; and ZY15–K88, *L. reuteri* ZY15 diet group treated with ETEC K88.

At genus levels (Figure 9A), *Alloprevotella*, *Blautia*, and *Eubactenum_coprostanoligenes_group* were significantly, positively correlated with mTORC1, LPS, and ROS levels and negatively correlated with ZO-1 and occludin levels (*p* < 0.05). *Bacteroides* showed significant positive correlations with mTORC1 and ROS levels but negative correlations with ZO-1 and occludin levels (*p* < 0.05). Moreover, *Akkermansia* was positively associated with MUC1, MUC2, ZO-1, and occludin levels but negatively correlated with LPS and mTORC1. *Escherichia_Shigella* showed positive correlations with ROS (*p* < 0.05). *Clostridia_UCG.014* showed negative correlations with ROS, DAO, mTORC1, mTORC2, HIF-1α, and RORγt. In further comprehensive data analyses, *L. reuteri* ZY15 protected the intestines from injury and influenced host health by enhancing gut microbiota stability and barrier integrity, also reducing inflammation via AKT/mTOR/HIF-1α/RORγt/IL-17 signaling (Figure 9B).

## 4. Discussion

In recent decades, despite significant reductions in diarrheal disease mortality rates in the pig industry, they remain the primary cause of morbidity and mortality among weaned piglets [1]. ETEC K88 infection usually impairs intestinal epithelial barrier integrity, which induces diarrhea in these animals [26]. *L. reuteri* SLZX19-12 was shown to protect the colon from infection by enhancing gut microbiota stability and barrier integrity and reducing inflammation [23]. Although *L. reuteri* enhances intestinal barrier function and modulates immune function, the underlying ETEC K88 mechanisms remain unclear. A crucial aspect of this study is the unique features of *L. reuteri* ZY15 compared to other strains of *L. reuteri* and its specificity for combating ETEC K88-induced diarrhea. While multiple *L. reuteri* strains have been reported to enhance intestinal health, ZY15 displays distinct properties such as acid tolerance, bile salt resistance, and the ability to inhibit ETEC K88 growth in vitro (unpublished data). This strain was isolated from healthy piglets with high resistance to weaning stress, suggesting its unique adaptation to host-specific gut environments. Therefore, we investigated *L. reuteri* ZY15 effects on intestinal barrier and inflammation and its action mechanisms in mice challenged with ETEC K88. By observing mouse growth and activity, mice gavaged with ETEC K88 showed significantly reduced body weights and food intake. From pathogenic bacterial count assays, *Enterobacteriaceae* numbers in feces from K88 mice were significantly increased. H&E staining also showed damaged intestinal tissues. Combined, these observations indicated that an ETEC K88-induced mouse model was successfully established [27]. Subsequently, *L. reuteri* ZY15 was used to investigate any palliative effects in these mice.

The chemical barrier has important roles in isolating the internal and external environments of the intestinal tract, lubricating intestinal mucosa, and inhibiting harmful substance entry into the intestinal lumen [28]. In this study, ileum V/C ratios were significantly decreased and inflammation scores significantly increased after ECTC K88 infection, indicating a significantly disrupted intestinal epithelium. These observations were consistent with a previous report [29] showing that ETEC K88 adhered to intestinal epithelium surfaces and produced endotoxins, leading to a disrupted intestinal mucosa. Interestingly, *L. reuteri* ZY15 pretreatment significantly alleviated mucosal lesions in ETEC-challenged mice. Specifically, pretreatment significantly restrained MUC2 deficiency in the ileum of K88-induced mice, suggesting that *L. reuteri* ZY15 protected mucus layers by secreting adequate MUC2 levels. This observation was similar to that of Zhou et al. [27] who reported that *Lactobacillus plantarum* BSGP201683 enhanced the intestinal barrier in mice infected with ETEC K88. The mucus layer is the first barrier that covers the intestinal epithelium, which limits interactions between luminal contents and tissue [30]. MUC2 is a glycosylated protein secreted by intestinal goblet cells and the most important component of the mucus layer [31]. Furthermore, Intestinal Epithelial Cells (IECs) and intercellular junctional complexes such as tight junction (TJ) proteins constitute the largest and most important physical barriers segregating host tissue and the external environment to maintain intestinal homeostasis [32]. Evidence now suggests that LPS exposure facilitates IEC loss and TJ barrier dysfunction, which may participate in intestinal epithelial barrier disruption [33]. We demonstrated that *L. reuteri* ZY15 pretreatment significantly blocked TJ protein downregulation, including occludin and ZO-1 in the ileum of K88-treated mice. This was similar to the findings of Lin et al. [34], who reported that β-defensin 118 significantly alleviated ETEC-induced disruption by improving ZO-1 protein localization in the intestinal epithelium. One way to assess intestinal permeability is to use LPS and DAO [35]. In this study, when compared with K88 mice, decreased serum LPS and DAO concentrations in ZY15 and ZY15-K88 mice confirmed more integrated barrier structures. Taken together, *L. reuteri* ZY15 protective effects against K88-induced intestinal barrier injury may be associated with mucus layer maintenance and improved TJ barrier function. Similarly, *L. reuteri* ZY15 beneficial effects on intestinal epithelium morphology and integrity may be associated with its antibacterial and anti-inflammatory properties, as bacteria-deprived endotoxins and inflammatory cytokines (i.e., IL-6 and TNF-α) disrupt the intestinal mucosa [36].

It is widely recognized that intestinal barrier dysfunction induced by ETEC K88 leads to pathogen penetration and inflammatory cell infiltration into the intestinal mucosa and the overproduction of pro-inflammatory mediators, which aggravate intestinal inflammatory status and further damage intestinal barrier integrity [37]. Therefore, inflammatory cytokine transcript levels were examined to assess inflammation severity. From our data, K88 significantly upregulated proinflammatory cytokine expression (e.g., *IL-1β*, *TNF-α*, *IFN-γ*) in the ileum, which possibly caused immune disorders in the ileum, exacerbated inflammation, and damaged ileum tissue. However, *L. reuteri* ZY15 significantly downregulated this expression, suggesting modulatory effects on host immune responses by promoting balanced responses, consistent with previous reports [38,39,40].

Also, *L. reuteri* ZY15 pretreatment significantly reduced IL-17 (inflammatory cytokine) levels in mice challenged with ETEC K88. By recruiting neutrophils, monocytes, and polymorphonuclear leukocytes, IL-17 disrupts intestinal TJ proteins, creates gaps between epithelial cells, and further damages the epithelial barrier to exacerbate intestinal inflammation, thus creating a vicious cycle [41,42]. Therefore, therapeutic strategies targeting IL-17 or its signaling pathways may be used to therapeutically treat certain intestinal diseases. RORγt is a master transcription factor for IL-17 and is explicitly expressed in Th17, γδT, and type 3 innate lymphoid cells in mice and humans [43]. Since dysregulated IL-17 levels are strongly linked to several human inflammatory diseases, the RORγt-IL-17 axis has become a major research focus. In RORγt-deficient mice, IL-17 production was greatly diminished and RORγt/RORα double-deficient mice completely lacked IL-17 production [44,45]. Recent cell-level studies reported that HIF-1α promoted IL-17 secretion through RORγt [46]. HIF proteins are key cell response determinants of hypoxia. In tumor cells, activated HIF-1 has key adaptive response roles to altered oxygen levels via the transcriptional activation of over 100 downstream genes which regulate key biological processes required for tumor survival and progression. Such genes are involved in glucose metabolism, cell proliferation, and migration and angiogenesis [47]. Decreased oxygen concentrations (hypoxia) are major stressors that generally subvert the life of aerobic species and are prominent in pathological states underpinning inflammation and bacterial infection [48]. Blanda et al. [46] reported that mTORC1 induced HIF-1α activation, which reprogramed ILC3 metabolism toward glycolysis and sustained RORγt expression. Considering that in the ZY15-K88 group, the DEGs have the largest proportion of participation in inflammation-related pathways, we further conducted a GO analysis on significantly regulated DEGs in inflammation-related pathways. We observed that K88 significantly upregulated proinflammatory cytokine expression (*HIF-1α*, *RORγt*, and *IL-17*) and also AKT, mTORC1, and mTORC2 levels in the mouse ileum, which potentially damaged the intestinal barrier in the ileum, exacerbating inflammation. However, *L. reuteri* ZY15 significantly downregulated this expression, consistent with the study of Han et al. [49], who reported that *L. reuteri* suppressed Colorectal Cancer Tumorigenesis (CRC) tumorigenesis by inhibiting IL-17 signaling. Previous research has established that other *L. reuteri* strains alleviate inflammation and improve intestinal health through diverse mechanisms, such as the secretion of antimicrobial metabolites and the modulation of host immune responses [50,51]. This study highlights the distinctiveness of *L. reuteri* ZY15 in mitigating ETEC K88-induced intestinal inflammation and barrier dysfunction. While many *L. reuteri* strains exhibit probiotic effects, ZY15 demonstrates unique properties, such as its ability to inhibit ETEC K88 proliferation and modulate the AKT/mTOR/HIF-1α/RORγt/IL-17 signaling pathway.

The gut microbiota comprises many different microbial communities that regulate a host’s immune system [52]. Given the close relationship between a modulated gut microbiota and inflammation, it is important to examine a host’s microbiota to validate the potential of an anti-inflammatory probiotic therapy [53]. Therefore, we analyzed the gut microbiota in mice using *16S rRNA* sequencing and found that ETEC K88 challenge disrupted the structural balance in the microbiota, with different treatments generating their own distinct microbial communities. These results were consistent with a previous report indicating that the microbiota balance among probiotic, symbiotic, and pathogenic microorganisms was broken by invasive antigens [54]. In our study, *L. reuteri* ZY15 significantly increased *Actinobacteriota* and *Verrucomicrobia* abundance at phylum levels. In the gut, the most represented *Actinobacteriota* are *Bifidobacteria*, which exert beneficial effects on the gut barrier due to prominent short chain fatty acid (SCFA) production [55]. *Verrucomicrobia*, which reside in the inner intestinal mucosa layer, generate SCFAs such as propionate and butyrate [56]. *L. reuteri* ZY15’s addition significantly increased *Akkermansia* and *Clostridia_UCG.014* abundance at genus levels. *Akkermansia* primarily promote intestinal barrier integrity, modulate immune responses, and suppress inflammation [57]. *Clostridia_UCG.014* also promote beneficial bacterial growth, inhibit harmful pathogen growth in the intestinal tract, improve immunity, and promote nutrient absorption, digestion, and absorption [58]. Therefore, mechanistically, in ETEC K88-induced mice, *L. reuteri* ZY15 putatively repaired the microbial barrier by regulating interactions between the gut microbiota and inflammatory pathways.

During the evolution process, intestinal tissue and the gut microbiota have formed stable mutualistic relationships and may constitute a symbiotic relationship [15]. The results of the Spearman correlation analysis between gut microbiota and ileum cytokine expression showed a greatly different relationship between the K88 group and the Con group and the ZY15-K88 and the K88 group. These data suggested that reduced HIF-1α, RORγt, and IL-17 levels may contribute to intact intestinal structures and reduce systemic inflammatory responses. Comparative transcriptomic analyses suggest that ZY15 uniquely regulates the AKT/mTOR/HIF-1α/RORγt/IL-17 pathway, a critical axis in modulating intestinal inflammation and barrier function under ETEC K88 challenge. Furthermore, its ability to enrich beneficial gut microbes such as *Akkermansia* and *Clostridia_UCG.014* highlights its tailored action regarding gut microbiota restoration, essential for overcoming ETEC-induced dysbiosis. In contrast to other strains of *L. reuteri*, ZY15′s dual action—restoring the microbiota and modulating host signaling pathways—makes it a strong candidate as a next-generation probiotic for ETEC-induced diarrhea in weaned piglets.

## 5. Conclusions

*L. reuteri* ZY15 protected the ileum from ETEC K88 infection by enhancing gut microbiota stability and barrier integrity and reducing inflammation induced by oxidative stress. *L. reuteri* ZY15 regulated intestinal inflammatory responses via the AKT/mTOR/HIF-1α/RORγt/IL-17 pathway and enhanced chemical, mechanical, and biological barrier integrity by stabilizing the gut microbiota upon ETEC K88 challenge. This pathway could provide a potentially promising therapy for treating ETEC K88-induced diarrhea in weaned piglets. These data highlight some of the mechanisms whereby *L. reuteri* ZY15 ameliorates ETEC K88-induced diarrhea in animals.

## Figures and Tables

**Table 1:** Primer sequences for real-time quantitative PCR.

Target	Primer Sequence (5′-3′)	Accession Number
*GAPDH*	F: GGGTCCCAGCTTAGGTTCAT R: CCCAATACGGCCAAATCCGT	NM_001289726.2
*IL-1β*	F: ATGCCACCTTTTGACAGTGATG R: TGTGCTGCTGCGAGATTTGA	NM_008361.4
*IFN-γ*	F: TAGCCTCACCGCCTATCACT R: CACCAACATGTGCGGTTTGT	NM_010511.3
*Occludin*	F: GCAATGACATGTATGGCGGAG R: TGTCCCAAGCAAGTGTGGAA	NM_001360537.1
*ZO-1*	F: AGGTGAAACTCTGCTGAGCC R: GCAAAAGACCAACCGTCAGG	NM_001163574.1
*Claudin-1*	F: GGCTTCTCTGGGATGGATCG R: TTTGCGAAACGCAGGACATC	NM_016674.4

## Data Availability

The original contributions presented in this study are included in the article. Further inquiries can be directed to the corresponding authors. The sequencing data generated in this study were deposited in Sequence Read Archive (SRA) with the SRA accession numbers PRJNA1182467, PRJNA1182406, and PRJNA1182124.
